# The Clinical Characteristics and Treatment of Patients With Autoimmune Glial Fibrillary Acidic Protein Astrocytopathy (GFAP‐A): A Retrospective Study of 29 Patients

**DOI:** 10.1002/brb3.71159

**Published:** 2025-12-31

**Authors:** Xuewei Yang, Lanling Jin, Chanhong Shi, Hongwei Yue, Hongfei He, Wenli Zhu, Ruili Wei

**Affiliations:** ^1^ Department of Neurology Yiwu Hospital Affiliated to Wenzhou Medical University, Yiwu Central Hospital Yiwu China; ^2^ Department of Neurology Pujiang County People Hospital Pujiang China; ^3^ Department of Neurology First Affiliated Hospital, Zhejiang University School of Medicine Hangzhou China

**Keywords:** GFAP, GFAP‐A, NGS, thyroid function, treatment

## Abstract

**Purpose:**

To investigate the clinical features, treatment, and outcome of patients with autoimmune glial fibrillary acidic protein astrocytopathy (GFAP‐A).

**Methods:**

Medical records and collected case data from the First Affiliated Hospital of Zhejiang University School of Medicine from November 2020 to May 2024 and retrospectively analyzed the clinical features, radiological findings, laboratory findings, treatment, and outcomes of patients with autoimmune GFAP‐A.

**Results:**

Twenty‐nine eligible patients were included, predominantly male (21/29), with acute onset in 17 patients (58.6%). Common clinical syndromes included encephalitis, meningoencephalitis, encephalomyelitis, meningoencephalomyelitis, and myelitis. Magnetic resonance imaging (MRI) of the brain revealed widespread lesions (21/28). Cerebrospinal fluid (CSF) pressure, CSF nucleated cell count, CSF protein levels, CSF chloride, serum thyroid dysfunction, and abnormal blood cytokine levels correlated with disease severity but were not associated with prognosis. There was no correlation between CSF glucose level, serum GFAP antibody titer, CSF GFAP antibody titer, ferritin levels, human herpesvirus 4 (HHV‐4) in the CSF, and disease severity or prognosis. No malignancies were detected in any patient before or after disease onset. Most patients (25/29) had favorable outcomes. Immunotherapy was effective for both the short‐ and long‐term prognosis of GFAP‐associated disease. Non‐pulse steroid therapy and pulse steroid therapy showed comparable efficacy, while monoclonal antibody therapy was also potentially effective for GFAP‐A.

## Graphical abstract

A retrospective analysis of clinical features, treatment, and outcome of 29 patients with autoimmune glial fibrillary acidic protein astrocytopathy (GFAP‐A). Cerebrospinal fluid (CSF) pressure, CSF nucleated cell count, CSF protein levels, CSF chloride, serum thyroid dysfunction, and abnormal blood cytokine levels correlated with disease severity but were not associated with prognosis. There was no correlation between CSF glucose level, serum GFAP antibody titer, CSF GFAP antibody titer, ferritin levels, human herpesvirus 4 (HHV‐4) in the CSF, and disease severity or prognosis. Most patients (25/29) had favorable outcomes. Immunotherapy was effective for both the short‐term and long‐term prognosis in GFAP‐A. Non‐pulse steroid therapy and pulse steroid therapy showed comparable efficacy, while monoclonal antibody therapy was also potentially effective.

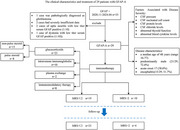

## 1 Introduction

Glial fibrillary acidic protein (GFAP) is an astrocyte cytoskeletal protein that plays a critical role in neuronal survival, axonal regeneration, and recovery of nervous system function. Under pathological conditions, the reactive expression of GFAP is significantly increased, and its elevated expression is a characteristic marker of astrocyte activation and proliferation (Mckeon and Benarroch 2018). Research on GFAP as a neurobiological marker has recently attracted widespread attention. As a reliable and noninvasive biomarker, it is highly valuable for assessing neurological damage and predicting prognosis (Abdelhak et al. 2022).

Autoimmune GFAP astrocytopathy (GFAP‐A) is a novel inflammatory disease of the central nervous system (CNS) reported by Fang in 2016 (Fang et al. 2016). Tissue‐based assays and cell‐based assays (CBAs) can be used to identify immunoglobulin G (IgG) reactive with GFAP in the cerebrospinal fluid (CSF) or serum of patients with GFAP‐A (Flanagan et al. 2017). The specific diagnosis of GFAP‐A is based on the presence of GFAP‐specific IgG in conjunction with the patient's clinical symptoms and examination results, while other conditions that may lead to positive GFAP antibodies, such as traumatic brain injury (Metting et al. 2012), dementia (Oeckl et al. 2022), and astrocytomas (Jung et al. 2007), are excluded. The clinical manifestations of GFAP‐A are predominantly encephalitis, meningoencephalitis, encephalomyelitis, meningoencephalomyelitis, and myelitis (Gravier‐Dumonceau et al. 2022; Iorio et al. 2018; Ke et al. 2024; Li et al. 2023; Long et al. 2018). Because the CSF shows increased lymphocyte predominance, with significantly elevated protein levels and decreased glucose and chloride levels, many patients are misdiagnosed with tuberculous meningitis in the early stages of GFAP‐A (Iorio et al. 2018). Magnetic resonance imaging (MRI) reveals a widespread distribution of lesions with linear gadolinium enhancement perpendicular to the lateral ventricles, which is a characteristic imaging finding of the disease but is not common. Although in Western countries GFAP‐A is often associated with tumors, this association has not been shown in northern or southern regions of China (Long et al. 2018), where GFAP‐A is more likely to be associated with infections. The exact etiology remains unclear and may be associated with both infections and tumors. Treatment for GFAP‐A includes high‐dose steroid therapy, intravenous immunoglobulin, and plasma exchange. Numerous studies have confirmed a good response to steroid treatment, and a meta‐analysis of 93 studies involving 681 patients revealed that corticosteroid treatment resulted in partial or complete remission in the majority of patients (83%) (Hagbohm et al. 2024). In recent years, monoclonal antibody therapy has gradually been applied to GFAP‐A. In summary, there are currently no definitive diagnostic criteria and no uniform treatment plan for GFAP‐A, and its pathogenesis has not been elucidated (Hagbohm et al. 2024).

With the continuous updating of literature data in recent years, GFAP‐A has gradually become recognized, and clinicians are more familiarized with it. The purpose of our study was to enrich the clinical symptoms, laboratory tests, and imaging findings and explore the optimal treatment plan for GFAP‐A, confirm the factors that influence the severity of the disease and prognosis, and discuss its pathogenesis.

## 2 Methods

### 2.1 Patient Selection

**FIGURE 1:**
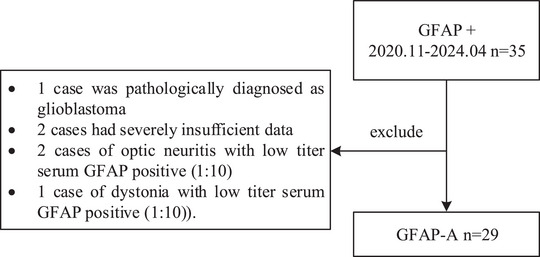
Consort diagram for patients included and excluded.

We reviewed the cases of patients with suspected neuroinflammatory diseases at the First Affiliated Hospital of Zhejiang University from November 2020 to May 2024 and found that 35 patients had tested positive for GFAP antibodies by CBAs. In this study, 29 cases were included, and 6 cases were excluded (Figure 1), of which 1 case was pathologically diagnosed with glioblastoma and 2 cases had severely insufficient data (1 case of encephalitis that was positive for serum GFAP (1:32) and 1 case of limb paresthesia that was positive for serum GFAP (1:32)). Moreover, to avoid false positives, three cases of non‐encephalitis with only low‐titer serum GFAP antibody positivity were excluded (2 cases of optic neuritis with low‐titer serum GFAP positivity (1:10) and 1 case of dystonia with low‐titer serum GFAP positivity (1:10)). The Modified Rankin Scale (MRS) was used to assess disease severity in the patients. MRS is an ordinal clinical scale ranging from 0 to 6 that quantifies functional disability or independent living capacity, and MRS scores ≤ 2 are considered a favorable outcome. This study was approved by the Ethics Committee of the First Affiliated Hospital of Zhejiang University School of Medicine (No. 2020‐IIT‐1163). All patients provided written or telephone informed consent.

### 2.2 Cell‐based Assay

The CBA method exhibits high specificity but reduced sensitivity due to precise antigen‐targeting capabilities and incomplete GFAP isoform coverage, consequently increasing false‐negative rates. In this retrospective study, TBA verification was selectively implemented in a subset of cases to increase detection rates. Given the absence of a unified positive titer threshold, empirically defined cutoffs (serum ≥ 1:10; CSF ≥ 1:1) were adopted on the basis of prior research consensus. Importantly, CSF positivity demonstrates superior diagnostic value over that of serum results owing to intrathecal antibody synthesis. Thus, serum‐only positivity has limited diagnostic utility and necessitates the integration of CSF profiles and imaging characteristics. To minimize false‐positive interference from sources such as blood‐brain barrier disruption or post‐infection cross‐reactivity, patients with serum titers ≤ 1:10 accompanied by non‐characteristic imaging findings were excluded.

The slides containing the antigen to be detected were removed, and the samples were allowed to equilibrate to room temperature. The working solutions (10% goat serum; 0.25% Triton X‐100) and wash solutions (phosphate buffered saline) and the required secondary antibodies (fluorescent secondary antibody (IgG) labeled with 488, diluted 1000‐fold) were removed, the mixture was equilibrated at room temperature, and it was mixed before use. The serum sample was diluted 1:10, and the CSF was not diluted. The slides were subsequently placed in a wet box, 200 µL of sample was added to each reaction zone, and the mixture was incubated at 37°C for 1 h. The primary antibody mixture was aspirated, and each well was washed with 200 µL of wash solution 3 times. For secondary antibody incubation, 200 µL of diluted FITC‐labeled IgG antibody was added to each well, and the samples were incubated for 30 min at room temperature. The washing was repeated. After glycerol was added, the samples were observed under a fluorescence microscope. When determining the titer, the serum was diluted at ratios of 1:100, 1:1000, and 1:10,000. Based on the intensity of the fluorescence signal, the antibody titer results are interpreted as follows: negative; 1:10; 1:32; 1:100; 1:320; 1:1000. For cerebrospinal fluid, it was diluted at ratios of 1:10, 1:100, and 1:1000. Based on the intensity of the fluorescence signal, the antibody titer results are interpreted as follows: negative; 1:1; 1:3.2; 1:10; 1:32; 1:100.

### 2.3 Statistical Analysis

Statistical analysis was performed with SPSS. The Wilcoxon signed‐rank test was used to compare the differences between the peak MRS scores, discharge MRS scores, and follow‐up MRS scores. Bonferroni correction was applied to correct the p‐values. The Spearman rank correlation test was used to analyze the correlations between the GFAP antibody titer, CSF pressure, CSF protein, CSF nucleated cell count, CSF glucose levels, CSF chloride levels, next‐generation sequencing (NGS), thyroid function abnormalities, ferritin levels, human herpesvirus 4 (HHV‐4) in the CSF, and disease severity and prognosis. The false discovery rate (FDR) method was applied to correct the *p*‐values. A *P* < 0.05 was considered statistically significant.

## 3 Results

### 3.1 Demographic Data

The demographics of the participants in this study are shown in Table 1. We included 29 patients with a median age of 48 years (range 16–77), who were predominantly male (21/29; 72.4%). The medical history of the 29 patients included hypertension (7 patients); prior tonsillectomy (1); a history of tuberculosis treatment (1); hyperthyroidism (1); diabetes (3); asthma (1); nephritis (1); cranial trauma (1); facial neuritis (1); and prostatic hyperplasia (1). With basic screening, no tumors were detected in any of the 29 patients.

**TABLE 1:** Characteristics of 29 patients with autoimmune GFAP astrocytopathy.

Characteristics	Patients, Number (%) (*n* = 29)
Age at onset (years‐old), median, range	48 [16–77]
Male	21 (72.4)
Past history	
Hypertension	7 (24.1)
Prior tonsillectomy	1 (3.4)
History of tuberculosis treatment	1 (3.4)
Hyperthyroidism	1 (3.4)
Diabetes	3 (10.3)
Asthma	1 (3.4)
Nephritis	1 (3.4)
Cranial trauma	1 (3.4)
Facial neuritis	1 (3.4)
Prostatic hyperplasia	1 (3.4)
Tumor	0 (0)
Mode of onset	
Acute onset	17 (58.6)
Subacute onset	5 (17.2)
Chronic onset	7 (24.1)
Clinical syndrome	
Encephalitis	15 (51.7)
Meningoencephalitis	5 (17.2)
Encephalomyelitis	4 (13.8)
Meningoencephalomyelitis	3 (10.3)
Myelitis	2 (6.9)
Clinical symptoms	
Common neuroinflammatory symptoms	
Fever	20 (69.0)
Headache	14 (48.3)
Tremor	16 (55.2)
Urinary and bowel dysfunction	16 (55.2)
Cognitive impairment	10 (34.5)
Disturbance of consciousness	9 (31.0)
Psychiatric abnormalities	9 (31.0)
Limb dysfunction	16 (55.2)
Seizure	11 (37.9)
Dizziness	8 (27.6)
Visual disturbance	1 (3.4)
Other symptoms	
Sleep disorders	8(27.6)
Sweating	1(3.4)
Palpitation	2 (6.9)
Cough with sputum	4 (13.8)
Loss of appetite	8 (27.6)
Nausea	9 (31.0)
Vomiting	10 (34.5)
Persistent hiccups	1 (3.4)
Abdominal distension	3 (10.3)
Diarrhea	1 (3.4)
Intestinal obstruction	2 (6.9)

### 3.2 Clinical Manifestations

The clinical manifestations of the 29 patients with autoimmune GFAP astrocytopathy are detailed in Table 1. Among them, 17 patients (58.6%) presented with acute onset, with symptoms peaking within 2 weeks; 5 patients (17.2%) presented with subacute onset, with symptoms gradually developing over 2 weeks to 3 months; and 7 patients (24.1%) presented with chronic onset, characterized by insidious symptom progression lasting more than 3 months. The most common initial symptoms were fever and headache. The most common clinical manifestations were fever (20; 69.0%), headache (14; 48.3%), tremor (16; 55.2%), urinary and bowel dysfunction (16; 55.2%), sleep disorders (8; 27.6%), cognitive impairment (10; 34.5%), disturbance of consciousness (9; 31.0%), psychiatric abnormalities (9; 31.0%), limb dysfunction (16; 55.2%), seizure (11; 37.9%), dizziness (8; 27.6%), and visual disturbance (1; 3.4%). Other symptoms included cough with sputum (4; 13.8%), sweating (1; 3.4%), palpitation (2; 6.9%), and gastrointestinal symptoms (17; 58.6%), including loss of appetite (8), nausea (9), vomiting (10), persistent hiccups (1), abdominal distension (3), diarrhea (1) and intestinal obstruction (2). Among these patients, one presented with abdominal distension as the initial symptom. The clinical syndromes included encephalitis (15/29; 51.7%), meningoencephalitis (5/29; 17.2%), encephalomyelitis (4/29; 13.8%), meningoencephalomyelitis (3/29; 10.3%), and myelitis (2/29; 6.9%). See Supplementary Table 1 for further details.

### 3.3 Laboratory Findings

#### 3.3.1 GFAP‐IgG Antibodies

Among all patients with positive serum or CSF samples, 15 were positive for GFAP antibodies in the CSF only, 9 were positive for both the serum and CSF samples, and 5 were positive for serum samples only. The median GFAP antibody titer in 24 CSF samples was 10 (range: 1–100), while the median titer in 14 serum samples was also 10 (range: 10–100). The CSF GFAP antibody titer did not correlate with disease severity (*p* = 0.688) or disease prognosis (*p* = 0.813). The serum GFAP antibody titer did not correlate with disease severity (*p* = 0.713) or disease prognosis (*p* = 0.860). Detailed information is provided in Supplementary Table 2.

#### 3.3.2 Routine Cerebrospinal Fluid Tests

All 29 patients underwent lumbar puncture for investigation. Seven of these patients presented to other hospitals in the early stages of their illness, where they underwent lumbar puncture and received appropriate symptomatic treatment before being transferred to the First Affiliated Hospital of Zhejiang University for further care. The remaining 22 patients underwent their first lumbar puncture at our hospital after the onset of symptoms. The detailed data are presented in Table 2. Most of the 22 patients had normal CSF pressure (median: 167.5 mmH_2_O; range: 20–350 mmH2O). Among these patients, 12 patients (54.55%) had normal CSF pressure (80–180 mmH_2_O), eight patients (36.36%) had increased CSF pressure, and two patients exhibited a decrease in intracranial pressure during the initial lumbar puncture. Additionally, five other patients, who did not show such a decrease initially, were found to have low intracranial pressure upon repeat lumbar puncture during treatment. In addition, 16 patients (72.73%) presented an elevated CSF nucleated cell count (normal range: 0–8 × 10⁶/L), with a median of 80 × 10⁶/L (range: 0–365 × 10⁶/L), predominantly consisting of lymphocytes. Notably, all six patients with normal nucleated cell counts presented with chronic manifestations. In addition, 17 patients (77.3%) had elevated CSF protein levels (normal range: 0.15–0.45 g/L), with a median of 1.35 g/L (range: 0.69–2.12 g/L). Among these patients, 12 (54.5%) had significantly elevated protein levels (>1.0 g/L). Seven patients (31.8%) had decreased CSF glucose levels (<2.5 mmol/L; normal range: 2.5–4.5 mmol/L), whereas 13 patients (59.1%) had decreased CSF chloride levels (<120 mmol/L; normal range: 120–131 mmol/L). See Supplementary Table 1 for further details. We analyzed the correlation between CSF results and peak MRS scores and at follow‐up. The analysis revealed a positive correlation between CSF pressure and disease severity (*p* = 0.018), with no significant correlation with prognosis (*p* = 0.523). In addition, the number of nucleated cells in the CSF was positively correlated with disease severity (*p* = 0.011), but there was no significant correlation with prognosis (*p* = 0.332). CSF protein levels were also positively correlated with disease severity (*P* < 0.011), while no significant correlation was found with prognosis (*p* = 0.359). Conversely, CSF chloride levels were negatively correlated with disease severity (*p* = 0.019), with no significant correlation with prognosis (*p* = 0.578). Finally, no significant correlations were observed between CSF glucose levels and either disease severity (*p* = 0.170) or prognosis (*p* = 0.925).

**TABLE 2:** Investigations of 29 patients with autoimmune GFAP astrocytopathy.

Investigations	Patients, Number (%) (*n* = 29)
CSF examination on admission	
CSF pressure, mmH_2_O, Median, range	167.5 [20–350]
CSF nucleated cell count, × 10⁶/L, Median, range	80 [0–365]
Elevated CSF protein levels, g/L	17 (77.3%)
Decreased CSF glucose level, mmol/L	7 (31.8%)
Decreased CSF chloride level, mmol/L	13 (59.1%)
Next‐generation sequencing	
Human herpesvirus 4	7 (46.7%)
Human herpesvirus type 5	1 (6.7%)
Streptococcus constellatus	1 (6.7%)
Mycobacterium marinum	1 (6.7%)
Common microbiome	1 (6.7%)
Other co‐occurring antibodies	
Anti‐neurofilament heavy chain	1 (3.4%)
Anti‐dynamin 1 antibodies	1 (3.4%)
Anti‐contactin‐associated protein 2	1 (3.4%)
Serum thyroid dysfunction	15 (65.2%)
Abnormal blood cytokine levels	5 (62.5%)
Brain MRI	28
Abnormal T2/FLAIR lesions	21 (75.0)
No abnormal T2/FLAIR lesions	7 (25.0)
Gadolinium‐enhanced brain MRI	18
Abnormal enhancement	7 (38.9)
No abnormal enhancement	11 (61.1)
Spine MRI	12
Abnormal T2/FLAIR lesions	6 (50.0)
No abnormal T2/FLAIR lesions	6 (50.0)
Gadolinium‐enhanced spine MRI	8
Abnormal enhancement	5 (62.5)
No abnormal enhancement	3 (37.5)
Electromyogram	9
Peripheral nerve damage	6 (66.7)
No significant abnormalities	3 (33.3)
Electroencephalography	22
Excessive delta and theta	15 (68.2)
Epileptiform discharges	3 (13.6)
No significant abnormalities	4 (18.2)

#### 3.3.3 Cerebrospinal Fluid Next‐generation Sequencing

NGS can theoretically detect all known pathogenic microorganisms with sequenced genomes. The detailed data are presented in Table 2. Fifteen patients underwent CSF NGS testing in the early stages of their disease. Among these patients, seven tested positive for human herpesvirus type 4 (for viral strains 1, 3, 3, 4, 5, 7, and 9, with one patient coinfected with human herpesvirus type 5 showing 6 strains). Five patients had negative test results, while the remaining three were identified as having other pathogens as follows: 1 case of Mycobacterium gordonae; 1 case of common microbiota, including Peptostreptococcus anaerobius, Lactobacillus gasseri, Streptococcus agalactiae, and Actinomyces; and 1 case of Streptococcus constellation.

Patients who tested positive by NGS appeared to have more severe disease, with one patient relapsing 1 year later and another dying shortly after discharge due to multiple organ failure; however, statistical analysis indicated no significant correlation with disease severity (*p* = 0.708) or prognosis (*p* = 0.272).

#### 3.3.4 Other Co‐occurring Antibodies

Three patients were found to have antibodies associated with other autoimmune diseases in their serum. One patient was positive for soluble nuclear protein antibodies and anti‐SSA52, and another had antinuclear antibodies, soluble nuclear protein antibodies, RNP, and anti‐PM‐SCL. All three patients had a peak MRS score of 4 during their illness, but all had a favorable outcome (MRS score of 1).

Additionally, CSF from three patients contained other neural antibodies; one patient had anti‐neurofilament heavy chain (anti‐NF‐H) and anti‐dynamin 1 (anti‐DNM1) antibodies, whereas two patients were positive for anti‐contactin‐associated protein 2 (anti‐CASPR2) antibodies. The detailed data are presented in Table 2. Among these patients, two had a peak MRS score of 5, and one had a peak MRS score of 1. Two of these patients had a good prognosis (with follow‐up MRS scores of 1 and 0), whereas one patient with anti‐CASPR2 fared poorly and died shortly after discharge due to multiple organ failure. Known coexisting antibodies, including those against the *N*‐methyl‐*D*‐aspartate receptor (NMDAR), myelin oligodendrocyte glycoprotein (MOG), aquaporin‐4 (AQP4), glutamic acid decarboxylase (GAD), and leucine‐rich glioma‐inactivated 1 (LGI1), were not screened for in this study.

#### 3.3.5 Thyroid Indicators

In this study, 23 patients underwent thyroid function tests during hospitalization. Among these patients, 15 had abnormal thyroid function. One had elevated thyroid function indices (with a history of hyperthyroidism), and 14 had hypothyroidism with decreased triiodothyronine levels (with decreased thyroid‐stimulating hormone levels in three patients and decreased free triiodothyronine levels in five patients). Thyroid peroxidase (TPO) levels were tested in 21 patients, and thyroid globulin (TG) levels were tested in 16 patients. Only one patient had a positive result, in whom both TPO and TG were present simultaneously, whereas the results of all other patients were negative. See Table 2 for details. The analysis revealed that patients with abnormal thyroid function had more severe disease (*p* = 0.011), but there was no significant correlation with prognosis (*p* = 0.288).

#### 3.3.6 Serum Immune Markers

Eight patients underwent extensive cytokine testing prior to immunotherapy. Five patients had abnormal cytokine results, while three patients had normal results. The detailed data are presented in Table 2. Analysis revealed that patients with abnormal cytokine levels had more severe disease (*p* = 0.044), but there was no significant correlation with prognosis (*p* = 0.440). Among the proinflammatory factors, IL‐6 was elevated in four patients, IL‐8 in one patient, IL‐12P70 in one patient, and IFN‐*γ* in one patient. Moreover, among the anti‐inflammatory factors, IL‐4 was elevated in three patients, and IL‐10 was elevated in one patient.

#### 3.3.7 Tumor Markers

Twenty‐five patients underwent comprehensive tumor marker testing at enrollment. The results revealed that one patient had a mild increase in squamous cell carcinoma‐associated antigen, and 13 patients had varying degrees of elevated serum ferritin levels. Among the five patients who were retested for ferritin after treatment, three returned to normal levels, whereas two patients did not return to normal ferritin levels. The analysis revealed no effect of ferritin levels on disease severity (*p* = 0.473) or prognosis (*p* = 0.391). As previously mentioned, none of the 29 patients was diagnosed with a tumor. However, we acknowledge that this retrospective study did not involve systematic cancer screening, such as chest‐abdominal CT or PET scans. Instead, the absence of cancer was assessed based on a combination of tumor markers, clinical manifestations, and follow‐up status during hospitalization. However, undetected cancer does not necessarily indicate its absence. We intend to address this issue through further prospective studies and also remind clinicians to remain vigilant about cancer screening.

### 3.4 Radiological Examination

**FIGURE 2:**
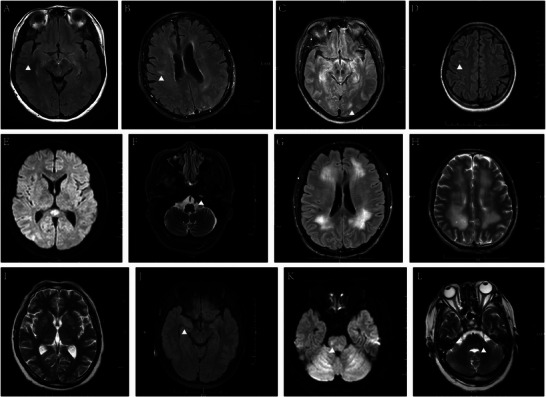
Different manifestations on cranial MRI in patients with GFAP‐A. **(A)** Swelling of the temporal lobe on the T2 flare sequence, **(B)** Patchy high signal in the right parietal lobe on the T2 FLAIR sequence, **(C)** Small nodular high signal in the left occipital lobe on the T2 FLAIR sequence, **(D)** Punctual abnormal signal in the right frontal lobe on the T2 FLAIR sequence, **(E)** The DWI sequence shows high signal intensity in the area of corpus callosum compression, **(F)** High signal intensity at the apex of the left temporal bone on the T2 sequence, **(G)** High signal intensity near the lateral ventricle on the T2 FLAIR sequence, **(H)** High signal intensity in the center of the semioval region on the T2 sequence, **(I)** High signal intensity in the basal ganglia region on the T2 sequence, **(J)** Hippocampal sclerosis on the T2 FLAIR sequence. **(K)** High signal intensity on the right side of the brainstem on the DWI sequence, and **(L)** High signal of the left pontine arm on the T2 sequence.

**FIGURE 3:**
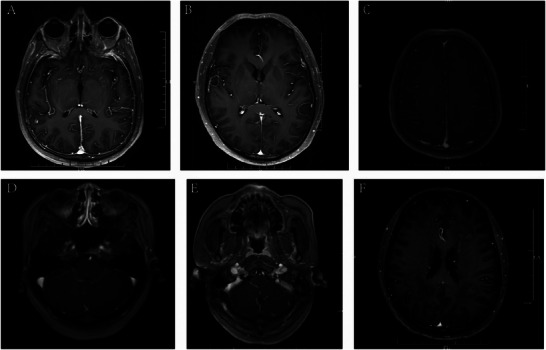
Enhanced cranial MRI shows enhancement of the dura mater, pia mater, paraventricular, and basal ganglia. **(A)** Significant enhancement of the dura mater; **(B–C)** Apparent enhancement of the pia mater in the cerebral hemisphere; **(D–E)** Apparent enhancement of the pia mater in the cerebellar hemisphere; and **(F)** Linear and patchy enhancement of the lateral ventricles bilaterally.

**FIGURE 4:**
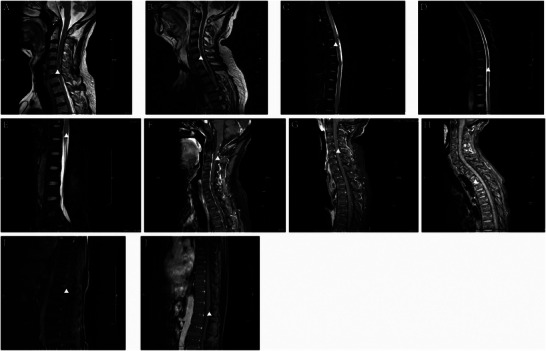
Different manifestations of the cervical, thoracic, and lumbar spinal cord on MRI. (**A–B)** The cervical spinal cord shows cord‐like and patchy high signal intensity on the T2 sequence; **(C–D)** The thoracic spinal cord shows linear, patchy, and patchy high signal intensity on the T2 sequence; **(E)** The lumbar spinal cord shows a slightly thickened spinal cord membrane on the T2 sequence; **(F)** The cervical spinal cord shows significant enhancement; and **(G–I)** The cervical, thoracic and lumbar spinal cords show different degrees of enhancement.

In patients with GFAP‐A, cranial MRI lesions were distributed in the temporal lobe (2/21), parietal lobe (2/21), occipital lobe (2/21), frontal lobe (1/21), splenium of the corpus callosum (3/21), centrum semiovale (1/21), lateral ventricles (1/21), basal ganglia region (1/21), insula (1/21), hippocampus (4/21), pons (4/21), medulla oblongata (1/21), middle cerebellar peduncle (1/21), pia mater (6/21), dura mater (1/21), cerebral peduncle (1/21), and temporal bone. (1/21) (Figure 2). Lesions in the paraventricular regions of the lateral ventricles and the semiovular nucleus often had a symmetrical distribution. More than one‐third of patients (7/18) presented with enhancing lesions on gadolinium‐enhanced cranial MRI. Symmetrical linear enhancement of the dura mater was revealed by MRI (Figure 3A), which also showed enhancement of the pia mater in the cerebral and cerebellar hemispheres (Figures 3B–E), linear and patchy enhancement in the lateral ventricles and centrum semiovale (Figure 3F), and enhancement of the brainstem. In addition, we found that five patients (5/8) had varying degrees of high signal intensity and enhancement changes in the cervical, thoracic, and lumbar spinal cord and meninges, and one patient's lumbar enlargement was slightly greater (Figure 4). Electroencephalogram (EEG) abnormalities were characterized mainly by increased power in delta and theta waves (15/22), with a few patients (3/22) showing epileptiform discharges. Peripheral nerve electromyography revealed peripheral nerve damage (6/9). See Table 2 for further details.

### 3.5 Treatment

#### 3.5.1 T**reatment During Hospitalization**

According to the diagnosis and treatment consensus for autoimmune encephalitis and the summary of our team's long‐term experience in managing GFAP‐A, all 29 patients received immunotherapy. See Table 3 for further details. A total of 23 patients were treated with glucocorticoids (15 patients received medium to high doses (equivalent to 20–120 mg/day methylprednisolone), and 8 received high‐dose therapy (500 mg/day for 3–5 days followed by a gradual taper). Ten patients were treated with intravenous immunoglobulin (IVIG), 2 underwent plasma exchange, and 8 received immunomodulatory therapy (1 on tacrolimus, 5 on rituximab, and 2 on efgartigimod). Notably, 1 patient who relapsed in this study did not respond to subsequent treatment with glucocorticoids and IVIG but showed significant improvement after receiving ofatumumab therapy (20 mg subcutaneous injection once weekly for 3 weeks).

**TABLE 3:** Therapy and outcome of 29 patients with autoimmune GFAP astrocytopathy.

	Patients, Number (%) (*n* = 29)
Therapy	
Glucocorticoids	23 (79.3)
Intravenous immunoglobulin	10 (34.5)
Plasma exchange	2 (6.9)
Immunomodulatory therapy	8 (27.6)
Outcome	
Unidirectional course	26 (89.7)
Relapsing and progressive course	2 (6.9)
Death	1 (3.4)
Modified Rankin Scale Score	
At admission, Median, range	4 [1–5]
At the peak of the disease, Median, range	4 [1–5]
At discharge, Median, range	2 [0–5]
At last follow up, Median, range	1 [0–6]
Hospitalization period, day, Median, range	21 [4–88]
Treatment interval, month, Median, range	2 [0–18]
Follow‐up period, month	14 [5–50]

#### 3.5.2 Treatment After Discharge

Of the 26 patients who continued immunotherapy after discharge, 15 were treated with oral corticosteroids alone, 5 received oral corticosteroids in combination with mycophenolate mofetil, 1 patient received oral corticosteroids in combination with tacrolimus, and 5 patients received regular rituximab injections (2–4 times, with an intravenous injection of 600 mg rituximab every 2 weeks to 1 month). Two patients discontinued immunotherapy after discharge, and one patient refused to continue treatment and died shortly after leaving the hospital.

### 3.6 Outcomes

**FIGURE 5:**
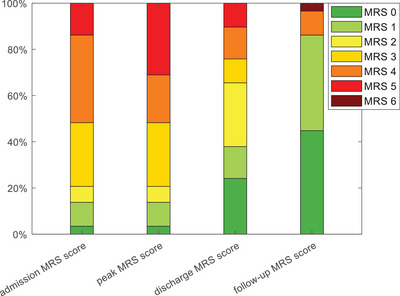
Grotta diagram of MRS at various time points.

Among the 29 patients, 24 exhibited identical MRS scores at admission (median 4; range 1–5) and at the peak of the disease (median 4; range 1–5). The median time for the MRS score to reach its peak (nadir) was 0 days, indicating that this disease has a rapid onset and that some patients may reach peak severity immediately. Figure 5 shows the grotta diagram of MRS at various time points. Follow‐up was performed on 29 patients, with a mean follow‐up of 20.2 months (median 14; range: 5–50 months). The average length of hospital stay was 22.83 days (median, 21; range: 4–88 days). See Table 3 for further details. A comparison of peak and discharge MRS scores (median 2; range 0–5) (p = 0.006) and follow‐up MRS scores (median 1; range 0–6) (P < 0.003) confirmed that immunotherapy had a favorable effect on both the short‐term and long‐term prognosis of GFAP‐A disease. Detailed information is provided in the Supplementary Table 3. In addition, a comparison of the results of the first lumbar puncture at disease onset and the last lumbar puncture after treatment in 15 patients revealed statistically significant differences in CSF pressure (*p* = 0.015), CSF nucleated cell count (P < 0.03), and CSF protein (P = 0.015), further confirming the efficacy of immunotherapy against GFAP‐A. Detailed information is provided in the Supplementary Table 4.

In this study, most patients (25/29) had a good prognosis (follow‐up MRS score of 1). Among the patients, 26 had a unidirectional course, 2 had a relapsing and progressive course, and one death was recorded. One of these patients relapsed after 11 months of maintenance therapy with oral steroids combined with tacrolimus, with new and more severe symptoms. High‐dose steroid therapy combined with intravenous immunoglobulin was ineffective, but treatment with ofatumumab (20 mg subcutaneous injection once weekly for 3 weeks) was effective (follow‐up MRS score of 1). Another patient, after 3 months of maintenance therapy with oral steroids combined with mycophenolate mofetil, experienced worsening of the same symptoms and relapsed but responded well to a second course of pulse steroid therapy (follow‐up MRS score of 1).

Owing to the limited number of cases, we chose to treat a larger number of patients for statistical analysis. There were 14 patients who received glucocorticoid therapy alone, 4 patients who received monoclonal antibody therapy alone, and the remaining patients received various combinations of two or three therapies. The analysis revealed that patients who received steroid therapy had more severe disease at the time of admission than did those who received monoclonal antibody therapy (*p* = 0.003, Z = ‐2.840). Potential explanations for these findings include treatment selection bias, where clinicians tended to prioritize steroid therapy for more severe cases while opting for monoclonal antibodies (mAbs) in mild‐to‐moderate patients, as well as differences in clinical indications. Specifically, our study revealed a preference for mAbs in chronic onset patients with relatively mild disease manifestations. This may be attributed to steroids' rapid anti‐inflammatory effects (albeit with potential immunosuppressive consequences) compared with the more targeted mechanism of mAbs, which results in a slower therapeutic onset. We also found that there was no difference in the admission MRS scores between patients receiving non‐pulse glucocorticoid therapy (10/14) and those receiving pulse glucocorticoid therapy (4/14) (*p* = 0.839). While 8 patients were receiving maintenance immunotherapy, 20 discontinued treatments. The average treatment interval was 3.8 months (median, 2; range, 0–18), and there was a significant difference between the MRS score at discharge and the MRS score during follow‐up (*p* = 0.024), indicating that GFAP‐A has a chronic recovery phase and that treatment during the recovery period is also very important.

## 4 Discussion

This study described the clinical characteristics of 29 patients with anti‐GFAP IgG autoantibodies and confirmed the presence of anti‐GFAP antibodies in the serum and CSF using a CBA. Articles published recently on GFAP antibodies indicate that autoimmune GFAP‐A is not uncommon and is increasingly recognized by clinicians. This study adds significantly to the existing published data on clinical patients with GFAP‐A.

In numerous published clinical studies, the median age of onset of autoimmune GFAP‐A in adults ranges from 43 to 54 years, with an incidence of up to 65% in male patients. This finding is consistent with the results of the present study, which revealed a significantly higher incidence rate in males than in females (72.4%) (Fang et al. 2016). Furthermore, some studies have shown that autoimmune GFAP‐A mainly manifests as acute or subacute onset, and there is an overlap of autoimmune antibodies, including NMDAR, MOG, and AQP4 (Dubey et al. 2018). Notably, some GFAP antibody‐positive patients also have tumors. However, in previous studies conducted in China and Japan, the number of patients with coexisting tumors was low or even zero. No tumor cases were detected in this study by means of simple screening, but studies in France and the United States reported a relatively high proportion of coexisting tumors, especially in the United States, where the proportion reached 34%. These findings suggest that there may be differences in the pathogenesis of GFAP‐A among different geographical regions and ethnic groups. Nevertheless, clinicians should continue to emphasize the importance of tumor screening in patients with GFAP‐A, especially within 2 years of disease onset (Cheng et al. 2023).

In our study, the most common initial symptoms of 29 patients with autoimmune GFAP‐A were fever and headache. The clinical syndromes of these patients may manifest as encephalitis, meningoencephalitis, encephalomyelitis, meningoencephalomyelitis, and myelitis. This finding is also in general agreement with those of previous studies (Gravier‐Dumonceau et al. 2022, Dubey et al. 2018). The main clinical manifestations included fever, headache, tremor, urinary and bowel dysfunction, sleep disorders, cognitive impairment, altered consciousness, psychiatric abnormalities, limb dysfunction, seizure, decreased vision, sleep disorders, sweating, palpitation, cough with sputum, loss of appetite, nausea, vomiting, persistent hiccups, abdominal distension, diarrhea, and intestinal obstruction. The clinical heterogeneity of autoimmune GFAP‐A is not surprising, as it resembles the various neurological manifestations observed in patients with other autoimmune or paraneoplastic syndromes associated with antibodies binding to intracellular neuronal antigens such as GAD65 or Hu (Lennon 2012).

In our study, samples from 15 patients were positive for GFAP antibodies in CSF, 9 patients had samples positive in both serum and CSF, and 5 patients had samples positive only in serum. We demonstrated that there was no significant correlation between GFAP antibody titers (in both CSF and serum) and disease severity (Luo et al. 2019). Similarly, other studies have shown no significant correlation between GFAP antibody titers in the blood/CSF and initial disease severity in pediatric GFAP‐A patients, with a mean age of 6.3 ± 0.6 years (Luo et al. 2019, Fang et al. 2021). At present, the mechanism behind this phenomenon is not clear. Therefore, further clinical and animal studies are needed to validate this conclusion.

CSF pressure was within the normal range in most patients with GFAP antibodies in our study. However, there was a general upward trend in protein levels and nucleated cell counts, accompanied by a decrease in chloride levels. Further statistical analysis revealed that CSF pressure, CSF protein levels, and the CSF nucleated cell count were positively correlated with disease severity but were not significantly correlated with prognosis. CSF chloride levels were negatively correlated with disease severity but not significantly correlated with prognosis. Inflammatory exudation leads to a significant increase in CSF protein content (e.g., globulins and fibrinogen). These negatively charged macromolecules induce Donnan effects (attracting cations while repelling anions), driving Cl^−^ back into the plasma and reducing the concentration of Cl^−^ in the CSF. The more severe the disease is, the greater the protein exudation and the stronger the Donnan effect, resulting in a more pronounced decline in Cl^−^. Additionally, Cl^−^ in the CSF is primarily secreted via active transport through the Na⁺‐K⁺‐2Cl^−^ cotransporter in the choroid plexus. Severe inflammation can impair choroid plexus function, reducing Cl^−^ transport. Concurrently, ventricular obstruction or fibrin blockage of arachnoid granulations can disrupt CSF circulation, further diminishing Cl^−^ retention and exacerbating its decline in concentration. This study confirmed the correlation between CSF pressure, CSF protein levels, CSF nucleated cell count, CSF chloride levels, and disease severity, providing an important reference for formulating clinical treatment strategies and assessing prognosis. However, the study sample included seven patients who did not present for the first time at an early stage of the disease and had previously been treated at other medical institutions. Owing to incomplete case records and limited investigator recall, the accuracy of disease assessment for these patients was relatively low. In addition, the results of lumbar punctures performed at outside hospitals for some patients could not be included in the statistical analysis because of a lack of pre‐ and posttreatment comparative data, which limited the completeness of the available data. Therefore, future studies need to be prospective and strictly screen patients who present for the first time after the onset of the disease to obtain more comprehensive and accurate data.

In addition, 15 patients underwent NGS testing of CSF early in the course of the disease. The results revealed that seven patients were positive for human herpesvirus 4 (HHV‐4), among whom one patient was also positive for human herpesvirus 5. This finding suggests that viral infections may play a triggering or promoting role in the pathogenesis of autoimmune GFAP‐A (Gravier‐Dumonceau et al. 2022), which is consistent with previous research findings (Handoko et al. 2019, Issa et al. 2020). This study revealed that NGS positivity had no significant impact on disease severity or prognosis. Future prospective studies with expanded sample sizes are warranted to further investigate this finding.

Eight of the 29 patients underwent cytokine testing prior to immunotherapy, and 4 of them had elevated levels of the proinflammatory cytokine IL‐6. Emerging evidence suggests that neuroinflammatory components beyond the immune system, including microglia, macrophages, chemokines, cytokines, and CD8+ T cells, may drive GFAP‐A pathogenesis. In particular, this mechanistic framework highlights the central role of cytokine networks in disease initiation and progression (Long et al. 2018). The results of our study also suggest that patients with abnormal cytokine levels have more severe disease. Whether this can be used as a predictive indicator of disease severity requires further validation in clinical trials. We observed a decreasing trend in triiodothyronine (*T*_3_) levels in 14 out of 15 patients with thyroid dysfunction. Previous studies have confirmed that *T*_3_ plays an essential role in the formation and maturation of oligodendrocytes and in myelin formation (Fernandez and Pirondi 2004, Fernandez et al. 2009). GFAP is a marker of astrocytes that support the regeneration of mature myelin and the survival of oligodendrocytes. However, sustained activation of the nuclear factor erythroid 2‐related factor 2 (Nrf2) pathway in astrocytes can interfere with the cholesterol biosynthesis pathway, leading to reduced oligodendrocyte survival and impaired myelin regeneration (Molina‐Gonzalez et al. 2023). It is not known whether this pathway leads to a decrease in *T*_3_, and further research is needed to demonstrate this association. However, whether autoimmune GFAP‐A leads to a further decrease in *T*_3_ by affecting pituitary function is currently unclear. In various animal models of demyelination, *T*_3_ supplementation has been shown to improve myelin regeneration, axonal protection, and maintenance of neurotransmission (Dell'Acqua et al. 2012, Franco et al. 2008). Our study also revealed, for the first time, an association between serum thyroid function abnormalities and disease severity but no significant association with prognosis. Therefore, more clinical data are needed to verify the relationships between supplemental *T*_3_ levels and disease severity and prognosis in clinical patients.

In the GFAP‐A patient population, cranial MRI revealed lesion distribution in the temporal, parietal, and occipital lobes; the corpus callosum; and the lateral ventricles, basal ganglia, meninges, insula, cerebral peduncle, and brainstem. Enhanced cranial MR can also reveal linear enhancement of the dura mater, leptomeningeal enhancement, symmetrical linear enhancement of the lateral ventricles, and punctate enhancement phenomena, and we also found that the spinal cord and meninges of the neck, chest, and waist segments of eight patients presented high signal intensity and varying degrees of enhancement on the T2 sequence, which is consistent with previous findings (Dubey et al. 2018). Some subjects had epileptiform discharge characteristics in their electroencephalograms, with high consistency between discharge sources and lesion sites in patients with GFAP‐A. Peripheral nerve electromyography revealed evidence of peripheral nerve damage, which may be related to the role of GFAP in immune‐mediated peripheral neuropathy. However, owing to the small number of patients tested, we were unable to fully determine the impact of EEG and peripheral neuropathy on disease severity and prognosis.

All patients in our study received immunotherapy with an average follow‐up of 20.2 months (range: 5–50 months). And the results indicate that immunotherapy showed significant efficacy in both the short‐term and long‐term outcomes of patients with autoimmune GFAP‐A. Fifteen patients received medium‐ to high‐dose glucocorticoid therapy, 8 patients received pulse corticosteroid therapy, 10 patients received immunoglobulin therapy, 2 patients received plasma exchange therapy, and 8 patients received immunomodulatory therapy. Among the patients, 26 had a unidirectional course, whereas 2 had a relapsing and progressive course. Although this study revealed that non‐pulsed therapy was as effective as pulsed therapy, further studies with a larger sample size are needed to confirm its effectiveness. If no significant differences in disease severity between the two groups of patients are found, the use of pulse‐dose glucocorticoids should be avoided to reduce the risk of severe side effects such as femoral head necrosis and hyperglycemia. In addition, all five patients treated with rituximab in this study experienced positive therapeutic effects. Owing to our small sample size, further clinical trials are needed to confirm whether immunotherapy and glucocorticoid therapy can achieve the same therapeutic effect in patients with autoimmune GFAP‐A or whether we can use immunotherapy directly to achieve clinical symptom relief in these patients. In addition, one case of GFAP‐A relapsed after treatment with glucocorticoids and IVIG, but the patient's clinical symptoms improved significantly after subsequent treatment with the anti‐CD20 monoclonal antibody ofatumumab. Ofatumumab is approved only for the treatment of multiple sclerosis, and as the current treatment duration is insufficient to fully demonstrate the long‐term efficacy and safety of ofatumumab in the treatment of GFAP‐A, further clinical studies are needed.

The study results also confirmed that the severity of the patient's condition at the peak of the disease is significantly correlated with treatment prognosis. These results provide strong evidence to support the selection of clinical treatment strategies.

Comprehensive analysis revealed that although the onset of autoimmune GFAP‐A is usually characterized by acute onset and severe conditions, the overall prognosis for patients with this disease is relatively optimistic. In this study, 25 out of 29 patients had a good prognosis, with 26 exhibiting a monophasic disease course and 2 exhibiting a recurrent and progressive disease course. Therefore, early recognition, diagnosis, and treatment of patients with GFAP‐A by clinicians are crucial and are expected to significantly improve treatment outcomes. This study not only expands the scope of clinical research on GFAP‐related disorders but also statistically confirms for the first time that immunotherapy is effective for both short‐term and long‐term prognosis in patients with GFAP‐A. Both conventional‐dose and pulse corticosteroid therapies have comparable efficacy, while monoclonal antibody therapy has a potential effect. Additionally, this study may promote the diagnosis of autoimmune GFAP‐A and the formulation of diagnostic criteria. However, as a retrospective single‐center study, there is inherent selection and recall bias. Moreover, 7 of 29 patients had incomplete or delayed initial assessments. We will conduct prospective, multicenter studies for further verification of our findings.

## Author Contributions

X. Y. conducted the literature review and drafted the manuscript. L. J., C. S., H. Y. and H. H. made substantial contributions to the conception and interpretation of the data. W. Z. and R. W. were responsible for critically revising the manuscript and gave final approval of the version to be published. All the authors contributed to the article and approved the submitted version.

## Funding

This research was supported by the Zhejiang Provincial Natural Science Foundation of China under Grant No. LBY22H200004.

## Ethics Statement

This study was approved by the Ethics Committee of The First Affiliated Hospital of Zhejiang University School of Medicine.

## Conflicts of Interest

The author declare no conflicts of interest.

## Supporting information



Supplementary Tables: brb371159‐sup‐0001‐SuppMat.docx

## Data Availability

The datasets used and analyzed during the current study are available from the corresponding author upon reasonable request.
