# Supplementary material for: The Clinical Characteristics and Treatment of Patients With Autoimmune Glial Fibrillary Acidic Protein Astrocytopathy (GFAP‐A): A Retrospective Study of 29 Patients
Source: Brain Behav. 2025 Dec 31;16(1):e71159. doi: 10.1002/brb3.71159 (PMC12755398; doi:10.1002/brb3.71159)
Supplement: Supplementary file 1 — Supplementary Tables: brb371159‐sup‐0001‐SuppMat.docx [file BRB3-16-e71159-s001.docx]

Supplementary Table 1 Demographic, laboratory and imaging findings; treatment; and outcomes of 29 patients with autoimmune GFAP-A

| Patient no. sex/age (years) | Clinical syndrome | CSF at the first (NCC/Pro/Glu/Chl) | Thyroid dysfunction | Coexisting neural autoantibodies | NGS results | Immunotherapy | MRS pre-Tx、/at discharge/at the last fu/,fu(mo) |
| --- | --- | --- | --- | --- | --- | --- | --- |
| 1. M/48 | meningoencephalitis | N/N/N/N | N | - | HHV-4(7) | IVGC(NS)  Tacrolimus | 3/2/1/16 |
| 2. F/35 | encephalitis | 180/1172/2.5/119 | + | - | - | IVGC(NS) | 4/1/0/43 |
| 3. F/44 | encephalitis | 144/760/2.4/105 | + | - | HHV-4 (1) | IVGC(NS) | 3/1/0/29 |
| 4. M/59 | meningoencephalitis | 90/1680/2.5/118 | N | - | Streptococcus constellatus | IVGC(NS) | 5/0/0/26 |
| 5. M/48 | meningoencephalomyelitis | 118/1150/3.8/103 | N | anti-NF-H  anti-DNM1 | HHV-4 (3) | IVGC(NS) | 4/4/1/10 |
| 6. M/77 | encephalitis | 160/1760/2.7/124 | + | - | - | IVGC(S)  IVIG Efgartigimod | 5/4/4/6 |
| 7. M/26 | encephalomyelitis | 220/2080/2.2/118 | + | anti-CASPR2 | HHV-4 (5) | IVGC(S)  IVIG  PE | 4/5/4/5 |
| 8. M/16 | encephalitis | 3/240/3.9/125 | - | - | common microbiome | Rituximab | 1/0/0/14 |
| 9. M/34 | encephalitis | 187/1800/2.5/108 | - | - | N | IVGC(NS) | 4/2/0/11 |
| 10. M/61 | encephalomyelitis | 100/690/5.4/132 | N | - | N | IVGC(S) | 3/2/2/12 |
| 11. M/42 | meningoencephalomyelitis | N/N/N/N | + | - | HHV-4 (3)  HHV-4 (6) | IVGC(S)  IVIG  Rituximab | 5/5/6/19 |
| 12. M/29 | encephalitis | 80/900/3.2/117 | N | - | N | IVGC(S)  IVIG  PE | 4/4/1/25 |
| 13. F/59 | encephalitis | N/N/N/N | + | - | mycobacterium marinum | IVGC(NS)  IVIG | 4/4/1/41 |
| 14. M/36 | meningoencephalitis | 98/1186/2.5/121 | - | - | - | IVGC(NS) | 3/2/0/32 |
| 15. M/58 | enchalomyelitis | 160/1830/2.8/120 | + | - | N | IVGC(NS) | 3/2/0/34 |
| 16. M/65 | encephalitis | 110/1350/3.1/120 | + | - | N | IVIG | 3/0/0/47 |
| 17. F/70 | encephalitis | 5//810/5.1/127 | + | - | N | IVIG  Rituximab | 4/3/1/10 |
| 18. M/65 | encephalomyelitis | 210/2120/1.8/116 | + | - | HHV-4 (5) | IVGC(NS)  IVIG | 4/5/4/32 |
| 19. M/57 | myelitis | 1/390/3.8/126 | N | - | N | IVGC(NS) | 3/3/1/13 |
| 20. M/66 | myelitis | 108/1430/3.1/106 | + | - | N | IVGC(NS) | 3/2/1/14 |
| 21. M/56 | meningoencephalitis | N/N/N/N | - | - | N | IVGC(S) | 4/3/1/50 |
| 22. M/59 | encephalitis | 90/800/3/117 | + | - | - | IVGC(S) | 5/2/1/7 |
| 23. F/29 | encephalitis | 0/340/3/127 | - | - | N | Efgartigimod | 1/0/0/12 |
| 24. M/55 | encephalitis | N/N/N/N |  | - | N | IVGC(NS)  IVIG | 2/0/0/17 |
| 25. F/26 | encephalitis | 2/220/2.8/126 | + | - | N | Rituximab | 2/2/0/23 |
| 26. M/31 | meningoencephalitis | 365/2100/3/109 | + | - | HHV-4 (9) | IVGC(NS)  IVIG | 4/1/1/7 |
| 27. F/46 | encephalitis | 3/430/3.2/131 | - | - | N | Rituximab | 0/0/0/9 |
| 28. M/47 | meningoencephalomyelitis | N/N/N/N | - | - | - | IVGC(NS) | 4/1/1/12 |
| 29. M/28 | encephalitis | N/N/N/N | - | anti-CASPR2 | - | IVGC(S) | 1/0/0/10 |

Abbreviations: anti-CASPR2= anti-contactin-associated protein 2 antibodies, anti-NF-H= anti-neurofilament heavy chain antibodies, anti-DNM1= anti-

dynamin 1 antibodies, C: cerebrospinal fluid. CSF = cerebrospinal fluid, HHV-4= Human Herpesvirus Type 4, F = female, FU,=follow-up, GFAP = glial fibrillary acidic protein, IVGC(NS)= intravenous glucocorticoids(non-shock), IVGC(S)= intravenous glucocorticoids (shock), IVIG = intravenous immunoglobulin, M = male, MRS = modified Rankin scale score, NMDAR = N-methyl-D-aspartate receptor, N=not available, NCC = nucleated cell count,/μL. PE= plasma exchange, pre-Tx= pretreatment, Pro=protein, g/L, Glu=glucose, mmol/L, Chl=chlorine, mmol/L, S=serum, s+c=both serum and cerebrospinal fluid.

Supplementary**Tables 2** The correlations between the GFAP antibody titer, CSF pressure, CSF protein, CSF nucleated cell count, CSF glucose levels, CSF chloride levels, next-generation sequencing (NGS), thyroid function abnormalities, ferritin levels, human herpesvirus 4 (HHV-4) in the CSF and disease severity and prognosis.

| Lab findings | disease severity | | prognosis | |
| --- | --- | --- | --- | --- |
|  | p | p. adj | p | p. adj |
| CSF pressure | 0.008 | 0.0176 | 0.333 | 0.523286 |
| CSF nucleated cell count | 0.002 | 0.011 | 0.151 | 0.3322 |
| CSF protein levels | <0.001 | <0.011 | 0.098 | 0.359333 |
| CSF glucose levels | 0.108 | 0.169714 | 0.757 | 0.925222 |
| CSF chloride levels | 0.007 | 0.01925 | 0.42 | 0.5775 |
| Serum GFAP antibody titer | 0.583 | 0.712556 | 0.782 | 0.8602 |
| CSF GFAP antibody titer | 0.625 | 0.6875 | 0.813 | 0.813 |
| abnormal thyroid function | 0.003 | 0.011 | 0.157 | 0.287833 |
| ferritin levels | 0.344 | 0.473 | 0.071 | 0.3905 |
| abnormal cytokine levels | 0.024 | 0.044 | 0.04 | 0.44 |
| NGS | 0.708 | 0.708 | 0.099 | 0.27225 |

Note: P-values were adjusted for multiple testing using the Benjamini-Hochberg false discovery rate (FDR) procedure. Significant results are defined as adjusted P < 0.05.

Supplementary**Tables 3 ​**A comparison of peak MRS scores, discharge MRS scores and follow-up MRS scores

|  | p | p. adj |
| --- | --- | --- |
| peak MRS VS discharge MRS | 0.002 | 0.006 |
| peak MRS VS follow-up MRS | <0.001 | <0.003 |
| discharge MRS VS follow-up MRS | 0.008 | 0.024 |

Note: P-values were adjusted for multiple testing using the Bonferroni method. Significant results are defined as adjusted P < 0.05.

Supplementary**Tables 4 ​**A comparison of the results of the first lumbar puncture at disease onset and the last lumbar puncture after treatment in 15 patients

|  | p | p. adj |
| --- | --- | --- |
| first VS last lumbar puncture CSF pressure | 0.005 | 0.015 |
| first VS last lumbar puncture CSF nucleated cell count | <0.01 | P<0.03 |
| first VS last lumbar puncture CSF protein levels | 0.005 | 0.015 |

Note: P-values were adjusted for multiple testing using the Bonferroni method. Significant results are defined as adjusted P < 0.05.
